# How Accessible Was Information about H1N1 Flu? Literacy Assessments of CDC Guidance Documents for Different Audiences

**DOI:** 10.1371/journal.pone.0023583

**Published:** 2011-10-25

**Authors:** Lisa P. Lagassé, Rajiv N. Rimal, Katherine C. Smith, J. Douglas Storey, Elizabeth Rhoades, Daniel J. Barnett, Saad B. Omer, Jonathan Links

**Affiliations:** 1 Department of Health, Behavior and Society, Johns Hopkins Bloomberg School of Public Health, Baltimore, Maryland, United States of America; 2 Department of Environmental Health Sciences, Johns Hopkins Bloomberg School of Public Health, Baltimore, Maryland, United States of America; 3 Department of International Health, Johns Hopkins Bloomberg School of Public Health, Baltimore, Maryland, United States of America; Duke-National University of Singapore Graduate Medical School, Singapore

## Abstract

We assessed the literacy level and readability of online communications about H1N1/09 influenza issued by the Centers for Disease Control and Prevention (CDC) during the first month of outbreak. Documents were classified as targeting one of six audiences ranging in technical expertise. Flesch-Kincaid (FK) measure assessed literacy level for each group of documents. ANOVA models tested for differences in FK scores across target audiences and over time. Readability was assessed for documents targeting non-technical audiences using the Suitability Assessment of Materials (SAM). Overall, there was a main-effect by audience, *F*(5, 82) = 29.72, P<.001, but FK scores did not vary over time, *F*(2, 82) = .34, P>.05. A time-by-audience interaction was significant, *F*(10, 82) = 2.11, P<.05. Documents targeting non-technical audiences were found to be text-heavy and densely-formatted. The vocabulary and writing style were found to adequately reflect audience needs. The reading level of CDC guidance documents about H1N1/09 influenza varied appropriately according to the intended audience; sub-optimal formatting and layout may have rendered some text difficult to comprehend.

## Introduction

On April 24, 2009, the World Health Organization (WHO) issued an alert about a novel strain of influenza. By 30 May, more than 214 countries and territories had reported laboratory-confirmed cases of pandemic H1N1/09 influenza, with over 18,138 deaths worldwide [1]. In the United States, the first case of the virus was reported on April 23 and, within the next month, 6,552 cases with 9 deaths confirmed [2].

During the outbreak, the U.S. Centers for Disease Control and Prevention (CDC) played a primary role in disseminating information about the rapidly evolving situation to local and state health departments, the news media, and the general public. This paper uses a health literacy framework to assess how well that information was tailored for different audiences in terms of literacy grade level and readability. With this information, health communication experts can refine their approaches to specific audiences in preparation for future pandemics.

Communication plays a critical role in public health and its importance is particularly heightened during times of crisis [3]. The timely and accurate delivery of information during a disease outbreak is important for ensuring an appropriate public health response, limiting the spread of illness, and attending to those who are already infected. According to WHO (2005) guidelines, a key communication priority is to convey accurate information about the nature of the disease and ways in which it is spread. Additional communication needs include describing who is at risk, the nature of risk, and what can be done to avoid exposure and manage illness [4]. Realizing these goals can be a considerable challenge at times of disease outbreak, typically characterized by uncertainty about the nature of the disease and the pathogen involved, ambiguity about response priorities, and public anxiety. In the weeks following the H1N1/09 outbreak, working in a climate of urgency and public anxiety, health officials were charged with the task of providing the public with accurate information as the pandemic was unfolding, despite incomplete knowledge of the nature of and risks associated with the virus.

The Healthy People 2010 report (under objective 11-2) reminds public health communicators to pay special attention to health literacy, defined as “the degree to which individuals have the capacity to obtain, process, and understand basic health information.” A high literacy level may render a health document inaccessible to people with limited reading skills. This is particularly important when communicating with the lay public, as the national average reading level of U.S. adults is between 6^th^–8^th^ grade [5].

In addition to literacy level, the readability of a document also affects information accessibility. Readability is influenced by the complexity of content, the writing style of print information, and document-level characteristics such as page layout, use of visual aids, and typography. Text that is “readable” makes information more accessible and useful by improving comprehension, retention, and reading speed [6].

Of the many sources of health information available to the public, CDC guidance documents are among the most important and reputable sources for health professionals and laypersons alike. For this reason, CDC guidance documents were selected for analysis as representative of the kind of information that is likely to be useful and influential during times of health crisis. The aim of this study was to assess the literacy level and readability of written communications about novel H1N1/09 influenza targeted to specific lay and professional audiences throughout the United States.

## Methods

### Data Collection

In this prospective study, we collected data during the first month of the H1N1/09 outbreak between April 28, 2009 and May 28, 2009. We monitored coverage of H1N1/09 influenza in real time on the English-language CDC webpage titled “H1N1 Flu (Swine Flu): Information for Specific Groups” (http://www.cdc.gov/H1N1FLU/update.htm), which classifies the documents according to their intended audience. We captured the full text of all posted guidance documents during that period.

### Assessment Instruments

We assessed documents according to one measure of literacy level and one measure of overall readability. In assessing literacy level, we used a Microsoft Word utility to calculate the Flesch-Kincaid (FK) grade level, which yields a score corresponding to a United States school grade reading level. Widely used in studies assessing health information available on the World Wide Web [7],[8],[9],[10],[11], the FK score takes into account the average number of syllables per word and average length of sentences in the document. We chose this measure because of its wide usage and its automated nature, which allowed us to assess all the documents through a uniform procedure.

The FK grade level score is calculated based on verbal content but not other factors (including layout, visual ease of reading, and presentation format) that can determine access and comprehensibility. In order to address this shortcoming, we also used the Suitability Assessment of Materials (SAM) instrument [12] to assess the overall readability of the guidance documents targeting two particular audiences: laypersons and those in secondary distributive institutions (e.g., educational institutions, businesses and employers, and travelers and the travel industry). This subset of CDC guidance documents was chosen for two reasons. First, our initial inspection of these documents indicated that the formatting and wording of documents intended for technical professionals and primary distributive institutions (e.g., state or tribal health departments) did not vary much by topic or across time. However, such variations were observed among the subset of documents targeting laypersons and secondary distributive institutions, suggesting that there had been efforts to tailor the presentation of specific topical information to these audiences. Second, it was thought that document readability would be a more critical predictor of engagement with and comprehension of the target information among laypersons and members of the secondary distributive audience.

Developed by Doak, Doak, and Root for evaluating patient education materials, the SAM score takes into account criteria such as layout, typography, graphics, and surrounding context; thus assessing the entire presentation of the document and its accessibility to an intended audience. Scores for each category range from 0 (Not Suitable) to 2 (Superior). Though the SAM was originally validated in a clinical sample [12] and was developed for and is widely-applied in coding paper documents [13], [14], [15], [16], [17], the instrument has been previously employed in studies assessing the readability of online health education materials [18], [19], [20]. To our knowledge, no modified versions of the SAM have yet been developed to capture the unique experience on reading online. In order to better characterize readability features relevant to the internet, we modified the tool to improve its application to Web-based documents. We removed factors that were directly related to reading on a printed page, such as high contrast between the typeface and the page and the glossiness of the paper. We also added an item capturing the inclusion of World Wide Web links in order to better reflect the online information-seeking experience. With these revisions, the modified SAM assessed the following content-level characteristics: inclusion of a statement of purpose, the scope of material covered, and inclusion of a summary statement. Literacy-related features assessed in the SAM included the reading level (as assessed using the Flesch-Kincaid score), writing style, vocabulary, inclusion of context when introducing new material. Document-level features included the layout and typography as well as the use of subheadings and “road signs” to guide the reader through the document. Lastly, the SAM assessed reader stimulation, including factors related to text interaction with the reader (e.g., the use of questions or cues to action), links to additional Web sources, and reader motivation (e.g., partitioning of subtopics into smaller sections). Two independent coders coded all the content, and discrepancies were resolved through discussion in order to reach 100% coding agreement. A full description of the coding criteria is summarized in Table S1.

### Data Analysis

We identified the target audience for each document based on its placement on the CDC website (http://www.cdc.gov/H1N1FLU/update.htm). CDC identifies 12 different audiences, some with subgroups: emergency shelters; health care providers; state, local, tribal and territorial health officials; laboratorians; pharmacists; parents and caregivers; educational institutions; community and faith-based organizations; people at high risk for flu complications; businesses and employers; travelers and the travel industry; and people in contact with pigs. We collapsed these groups into six broad categories: technical personnel (health care providers, laboratorians and pharmacists); primary distributive institutions (state, local, tribal, and territorial health officials); secondary distributive institutions (educational institutions, businesses and employers, and travelers and the travel industry); laypersons (parents and caregivers, people at high risk for flu complications and people in contact with pigs); media (documents downloaded from the “press updates” page); and multiple audiences. Materials in this last category consisted of general reports and fact sheets. Within each category, documents were classified further by the date of original release in three time periods: first week after the outbreak, second week after the outbreak, and thereafter.

### Hypotheses

We proposed three hypotheses. First, we expected the FK grade level scores to decrease with progression through the audience chain, such that the scores would be highest for the technical audiences and lowest for laypersons. This was based on the reasoning that information meant for the consumption of technical audiences would contain more professional or scientific jargon and technical information, as compared to information meant for the consumption of lay audiences. Second, we expected the FK grade level scores to decrease over time. Because translating highly technical language into simpler forms takes more time and effort, we reasoned that, during the initial phase of the outbreak, the urgency to disseminate information quickly would likely outweigh the need to spend more time prioritizing information and refining the language. Over time, however, the language would become simpler as more resources are brought to bear and communication efforts become more refined. Lastly, we expected that there would be a time-by-audience interaction effect, such that reading level would decrease over time more rapidly for audiences further down in the audience chain (e.g., the laypersons and media) than for audiences further up in the audience chain (e.g., technical and primary distributive audiences).

Next, we used the SAM tool to assess the subset of documents targeting secondary distributive institutions and laypersons. We compared the absolute and mean scores for each SAM category, highlighting areas of strength and weakness in the writing and presentation of information for these two distinct audiences. The application of the SAM was intended to provide insight and context for the FK grade level scores.

## Results

### Literacy

During the study period, a total of *N* = 101 unique guidance documents were published on the CDC website and were captured for this analysis. Of these, 22 were identified as targeting technical audiences, 6 as targeting primary distributive institutions, 15 as targeting secondary distributive institutions, 27 as targeting laypersons, 13 as targeting media, and 18 as targeting multiple audiences (including those for whom particular audiences could not be determined).

**Figure 1:**
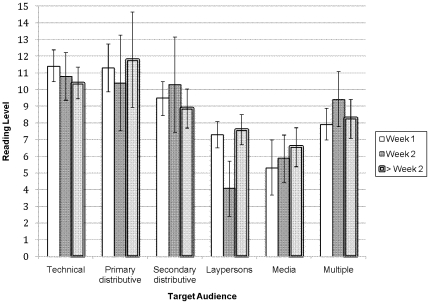
Flesch-Kincaid grade level scores of CDC guidance documents across audience types and over time (mean, 95% CI).

The average FK grade level score for all documents in our sample was 8.57 (*SD* = 2.31), corresponding approximately to a ninth-grade reading level. The FK grade level scores across audiences and over time are shown in Figure 1. Across the audience types, the highest grade level score was observed in documents targeting the primary distributive institutions (*M* = 11.2, *SD* = .62) and the lowest score across the entire time period was observed in documents targeting the media (*M* = 6.05, *SD* = .67). Overall, there was a significant main-effect of audience type, *F*(5, 82) = 29.72, P<.001. Grade level scores in documents targeting primary distributive institutions did not differ from those targeting technical audiences (*M* = 10.9, *SD* = 1.76), but both of these were significantly higher than the grade level scores targeting the other audiences. The grade level scores in documents targeting the media were significantly lower than those targeting the other groups.

Contrary to our expectations, grade level scores did not vary over time: *F*(2, 82) = .34, P>.05 on the FK grade level scores). There was, however, a significant audience × time interaction effect (*F*(10, 82) = 2.11, P<.05), the patterns of which are shown graphically in Figure 1. The grade level scores did not follow a discernible pattern across the different groups over time, but a post-hoc analysis revealed that the significant interaction was due to the patterns of means pertaining to laypersons. In particular, grade level scores of guidance documents targeting laypersons during the second week were significantly different from those targeting laypersons during the first week (*Z* = 3.23, P<.01) and those during the subsequent weeks of the study (*Z* = 3.53, P<.001). The low grade level score observed for laypersons during the second week was, in fact, significantly lower (all *Z*'s>1.96 and P's<.05) from all other 16 scores, except for two: documents targeting the media during the first week and the second week (for which the scores were not significantly different). The overall ANOVA model testing the effects of audience type, time, and audience × time interaction on FK grade level scores was significant, *F*(17,82) = 10.25, P<.001.

### Readability

The SAM tool was used to evaluate the readability of documents targeting secondary distributive institutions and laypersons in order to assess their readability. Scores for each of the readability criteria are presented in Table 1. Among documents targeting secondary distributive institutions (including, for example, local public health departments), three content-level factors emerged as areas of particular strength: the inclusion of a consistent and clearly defined statement of purpose (SAM score = 1.29); limiting the scope of information provided to only that pertaining to the stated purpose (SAM score = 2.0); and the appropriate use of Web links, guiding the reader to additional resources (SAM score = 1.93). One area requiring improvement was the inclusion of a summary statement. None of the documents in this sample included a summary statement to help the reader integrate and act on the information provided in the guidance document. Another area of considerable weakness among documents targeting secondary distributive institutions was the absence of rhetorical techniques that encourage interaction with the reader, such as posing questions and suggesting specific actions (SAM score = 0.07).

**Table 1:** Average SAM scores for CDC guidance documents targeting laypersons (n = 14) and secondary distributive institutions (n = 14).

	Laypersons	Secondary Distributive Institutions
**Content**		
Purpose	1.53	1.29
Scope	1.4	2.0
Summary	0.0	0.0
**Literacy**		
Reading Level	1.43	0.36
Writing Style	0.53	0.86
Vocabulary	0.53	0.43
Context	0.93	1.71
**Layout and Typography**		
Road Signs	1.73	1.57
Layout	0.0	0.0
Typography	1.07	1.0
Subheadings	0.13	0.29
**Stimulation**		
Interaction	0.53	0.07
Links	1.0	1.93
Motivation	0.80	0.86

Among those factors specifically assessing literacy levels, writing style was found to be acceptable among this sample of documents (SAM score = 0.86), as defined by the scoring criteria developed by Doak, Doak, and Root (1996) [12]. However, the complexity of vocabulary may have been too high in guidance documents targeting secondary distributive institutions (SAM score = 0.43), consistent with the higher reading grade level for this set of documents (FK = 9.5). Lastly, these documents require much improvement in their visual design and layout (SAM score = 0), meaning that they included none of the design elements that tend to improve readability. Similarly, these documents relied heavily on paragraph format, neglecting to break complex information down into more manageable chunks, such as in the form of bulleted lists (SAM score 0.29).

As with documents targeting secondary distributive institutions, two areas of particular strength among guidance documents aimed at the lay audience were the inclusion of a clearly defined statement of purpose (SAM score = 1.53); information presented in the documents was limited in scope consistent with the stated purpose (SAM score = 1.4). Additionally, these documents were strong in their use of advance organizers such as headers and topic captions (SAM score = 1.73), making the text appear more organized and allowing the reader to cognitively orient to the topic ahead. However, other positive aspects of visual presentation on the webpage were lacking. As with documents aimed at secondary distributive institutions, those targeting laypersons scored low on layout and the use of “chunking” (SAM scores = 0.0 and 0.13, respectively). As a result, although the reading level of this subsample of documents was appropriately geared towards a lay audience, the layout and organization of the text may have made these documents relatively difficult to process. Additionally, none of the documents in this subsample included a summary statement recapitulating the key messages, thus reducing the potential for retention and providing relatively weak encouragement for readers to act on the information.

## Discussion

It is important for health communication professionals to optimize the accessibility of information they provide to their audiences. This takes on added importance during a time of crisis, which is often marked by a rapidly evolving situation, a relative paucity of reliable information and heightened levels of anxiety and uncertainty. In our assessment of CDC guidance documents for novel H1N1/09 influenza, we identified six audience segments, and we assessed message tailoring across these segments and over time. We relied on the Flesch-Kincaid (FK) score to measure accessibility of content and the Suitability Assessment of Materials (SAM) score to assess presentation format.

We found that the reading level of CDC guidance documents about novel H1N1/09 influenza varied appropriately according to the intended audience. This conforms to basic tenets of risk communication that argue for matching audience needs with message components [21]. FK grade level scores for guidance documents aimed at the lay audience reflected the national average reading grade level. Contrary to our expectations, we did not observe variations in reading level over the month-long period following the outbreak. We had hypothesized that FK scores would decrease over time, demonstrating progressive prioritization and refinement of scientific and technical information for all audiences. Although we did not observe changes in reading level over time, we did observe an interaction effect between time and audience on the FK grade level. Documents directed at laypersons were written at higher levels at the beginning, but this decreased during the second week, and then increased again in the third and fourth weeks after the outbreak. This may reflect the difficulty of crafting simple messages (and hence the high reading level) during the initial outbreak period, followed by efforts to simplify language over time. The return to higher levels of reading difficulty during the later period may reflect the belief, on the part of communication experts, that audiences learn over time and thus can handle more difficult material as they become familiar with the topic and vocabulary, or it may reflect a belief that overly simplified language conveys less useful information. While currently speculative, this is worthy of further exploration, perhaps through discussion with the communication professionals at CDC who were responsible for preparing these guidance documents.

Analysis of the SAM scores revealed a number of challenges. Documents targeting secondary distributive institutions and lay audiences scored poorly on two presentation formats: layout and use of subheadings. Layout was scored on the basis of five characteristics: appropriate use of illustrations, consistency, use of visual cues to draw attention to key points, adequate white space, appropriate use of color, and short line length. Overall, documents were text-heavy with little use of visual cues and graphics, ignoring the maxim that “one picture is worth a thousand words.” Documents also used subheadings sparingly and chunking of text was minimal for lengthy sections. In contrast, both sets of documents received adequate scores for vocabulary and writing style, suggesting a tighter fit between verbal presentation of informational content and target audience needs. However, inadequate white space on the page, long lines of text, and a lack of visual cueing devices such as color, shading, or illustration, may have rendered the text more difficult to process and comprehend, regardless of the reading level.

Given the rapidly evolving nature of the emerging pandemic, real-time data capture was a key strength of this study. By collecting the complete set of CDC guidance documents as they were issued, this study presents a comprehensive picture of the information environment in the U.S. during the first month of novel H1N1 influenza outbreak. We are thus able to characterize the state of outbreak communication practices in the U.S. at the time of the initial outbreak of novel H1N1/09 virus. Overall, it appears that CDC guidance documents were tailored appropriately for the particular audience segments. Reading levels were highest for the technical and primary distributive audiences, groups that are likely to have a rich technical background for processing scientific information. Lowest grade level scores appeared in text written for the media. This may reflect a relatively sophisticated understanding of journalistic practices and imperatives. Common principles of effective media relations dictate that journalists are more likely to write about subjects if they are given information that requires less additional research or rewriting on their part. Indeed, much of the CDC content directed toward the media was written in the manner of press releases that could be used with minimal editing. The “rip and read” newsroom culture^8^ may have thus informed these Internet-based communications.

Although reading level is an important factor in ensuring accessibility of written health information, the formatting and presentation of written documents also deserves greater attention. Recognizing that reading speed diminishes online, handbooks on Web formatting mirror the SAM readability criteria, suggesting the use of simple, clear, and consistent language, simple syntax, and precise sentence structure throughout. In addition, Web designers are advised to present information in short passages, rather than long paragraphs, and to “chunk” text into bullets and short sentences grouped under topical headings. Lastly, Web designers are suggested to provide a clear organizational structure to guide the reader through the document and to provide links to outside documents, but do so parsimoniously [22], [23]. Better adherence to these recommendations on the part of public health professionals would likely improve both information dissemination efforts as well as public health response.

### Limitations

A number of limitations to this study require acknowledgment. First, while some tools exist to assess the usability of Internet documents, there are no resources available to specifically assess their readability. As a result, the research team employed the SAM, a tool developed for use with printed materials. Even so, we considered the SAM to be sufficiently suited to the online reading experience as it is possible that it would be even more difficult to read dense, poorly-formatted material on a small computer screen versus a larger page. Several earlier studies indicate that the material read on a screen is more difficult to understand and process than print material [12]–[14]. Consequently, the SAM provides a conservative estimate of online readability. Furthermore, no data were available on whether documents were read online or printed and read on paper; considering the possibility that documents may have been printed and posted (for instances in schools or workplaces), the SAM is an excellent option for assessing a potential variety of reading experiences.

Beyond inclusion on the CDC website, it is not known if and how information about novel H1N1/09 virus was distributed and to whom. The research team was not able to sample guidance documents that might have been sent directly to target audiences rather than (or in addition to) being posted online. It is possible that such documents may have varied in format, content, and readability from those available online. Nevertheless, given the prominence of the CDC website in conveying health information, we deemed it important to understand the nature of this communication. Further investigation would be needed to examine precisely how documents are distributed. Additional research is also needed to explore how members of the public access and use information in the time of an outbreak, and the extent to which they rely on and trust content received through the Internet compared to other sources.

### Conclusion

This study demonstrates an effort on the part of the U.S. public health system to tailor written health information about novel H1N1/09 virus to the needs of specific audiences by adjusting the reading level, vocabulary, and writing style. However, reading level alone does not determine information accessibility. Findings from the SAM assessment of CDC guidance documents about H1N1/09 influenza also demonstrate a lack of attention to visual and layout features that can improve the readability of health information. Public health communication should be crafted to reflect the informational needs as well as the technical capacity of the target audience. Further, the formatting of health communication documents should be formatted to improve accessibility, regardless of the target audience. This includes using a medium to large size font, allowing for adequate white apace on the page, limiting sentence length, and grouping bits of text to allow for easier navigation of the document. Future efforts should be made to ensure that all appropriate readability factors are considered when designing vitally important communication materials following the onset of an outbreak, when urgency and uncertainty are high and information needs are greatest.

## Supporting Information

Table S1 **Summary of SAM coding criteria, adjusted for use on web documents.**
(TIFF)
