# Correction: How Accessible Was Information about H1N1 Flu? Literacy Assessments of CDC Guidance Documents for Different Audiences

**DOI:** 10.1371/annotation/dfe07e51-a035-4eed-93a7-602a829cb49c

**Published:** 2011-11-18

**Authors:** Lisa P. Lagassé, Rajiv N. Rimal, Katherine C. Smith, J. Douglas Storey, Elizabeth Rhoades, Daniel J. Barnett, Saad B. Omer, Jonathan Links

The corrected funding statement is: "This research is funded by the Johns Hopkins Preparedness and Emergency Response Research Center (PERRC) through the Centers for Disease Control (CDC) grant no. P01TP000288 and partially funded by the Emory University PERRC through CDC grant no. 5P01TP000300. The funders had no role in the study design, data collection, analysis, decision to publish, or preparation of the manuscript."

