# Supplementary figures and images for: How Accessible Was Information about H1N1 Flu? Literacy Assessments of CDC Guidance Documents for Different Audiences
Source: PLoS One. 2011 Oct 25;6(10):e23583. doi: 10.1371/journal.pone.0023583 (PMC3201941; doi:10.1371/journal.pone.0023583)

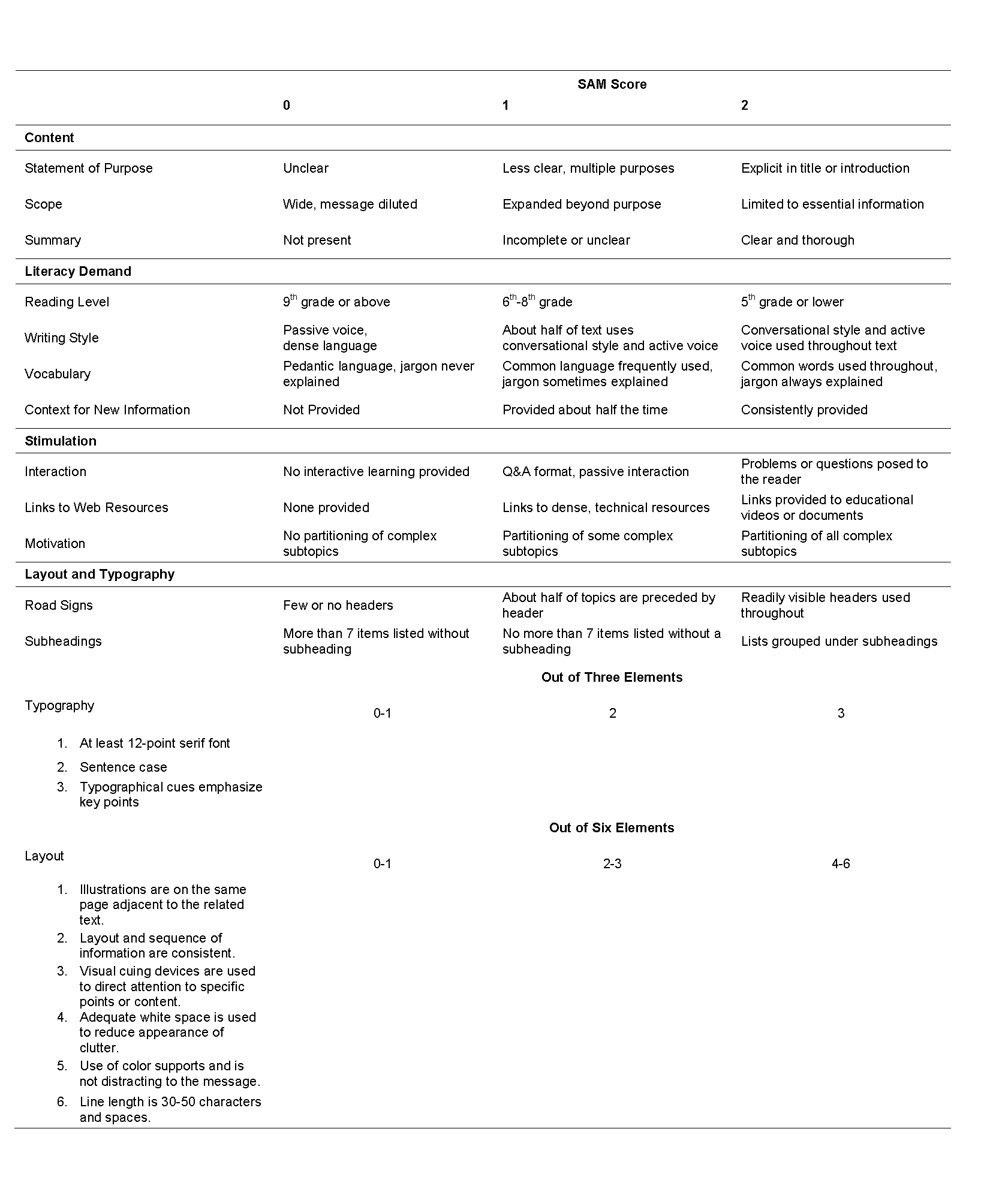

Supplement: Table S1 — Summary of SAM coding criteria, adjusted for use on web documents. (TIFF) [file pone.0023583.s001.tiff]
